# *Wolbachia* Infections Are Virulent and Inhibit the Human Malaria Parasite *Plasmodium Falciparum* in *Anopheles Gambiae*

**DOI:** 10.1371/journal.ppat.1002043

**Published:** 2011-05-19

**Authors:** Grant L. Hughes, Ryuichi Koga, Ping Xue, Takema Fukatsu, Jason L. Rasgon

**Affiliations:** 1 The W. Harry Feinstone Department of Molecular Microbiology and Immunology, Bloomberg School of Public Health, Johns Hopkins University, Baltimore, Maryland, United States of America; 2 The Johns Hopkins Malaria Research Institute, Baltimore, Maryland, United States of America; 3 National Institute of Advanced Industrial Science and Technology (AIST), Tsukuba, Japan; Stanford University, United States of America

## Abstract

Endosymbiotic *Wolbachia* bacteria are potent modulators of pathogen infection and transmission in multiple naturally and artificially infected insect species, including important vectors of human pathogens. *Anopheles* mosquitoes are naturally uninfected with *Wolbachia*, and stable artificial infections have not yet succeeded in this genus. Recent techniques have enabled establishment of somatic *Wolbachia* infections in *Anopheles*. Here, we characterize somatic infections of two diverse *Wolbachia* strains (wMelPop and wAlbB) in *Anopheles gambiae*, the major vector of human malaria. After infection, wMelPop disseminates widely in the mosquito, infecting the fat body, head, sensory organs and other tissues but is notably absent from the midgut and ovaries. *Wolbachia* initially induces the mosquito immune system, coincident with initial clearing of the infection, but then suppresses expression of immune genes, coincident with *Wolbachia* replication in the mosquito. Both wMelPop and wAlbB significantly inhibit *Plasmodium falciparum* oocyst levels in the mosquito midgut. Although not virulent in non-bloodfed mosquitoes, wMelPop exhibits a novel phenotype and is extremely virulent for approximately 12–24 hours post-bloodmeal, after which surviving mosquitoes exhibit similar mortality trajectories to control mosquitoes. The data suggest that if stable transinfections act in a similar manner to somatic infections, *Wolbachia* could potentially be used as part of a strategy to control the *Anopheles* mosquitoes that transmit malaria.

## Author Summary

Infection with *Wolbachia* bacteria has been shown to reduce pathogen levels in multiple mosquito species. *Anopheles* mosquitoes (the obligate vectors of human malaria) are naturally uninfected with *Wolbachia*, and stable artificial infections have not yet succeeded in this genus; however somatic infections can be established that can be used to assess the effect of *Wolbachia* infection in *Anopheles*. Here, we show that infection with two different *Wolbachia* strains (wMelPop and wAlbB) can significantly reduce levels of the human malaria parasite *Plasmodium falciparum* in *Anopheles gambiae*. After infection, *Wolbachia* disseminate throughout the mosquito but are notably absent from the gut and ovaries. The mosquito immune system is first induced in response to *Wolbachia* infection, but is then suppressed as the infection progresses. The *Wolbachia* strain wMelPop is highly virulent to *Anopheles* only after blood feeding. If stable infections can be established in *Anopheles*, and they act in a similar manner to somatic infections, *Wolbachia* could potentially be used as part of a strategy to control malaria.

## Introduction

Bacterial associates are ubiquitous among insects, including mosquitoes [1]. *Wolbachia* are obligate endosymbiotic bacteria that infect numerous insects, many of which are vectors of pathogenic microorganisms. Much interest has centered around *Wolbachia* as a means of reducing arthropod-borne disease due to the capacity of the bacteria to manipulate the reproduction of the insect host, which in turn favors their own transmission [2], [3]. However, recent studies detail that *Wolbachia* can directly cause pathogen interference (PI) in their invertebrate hosts, whereby infected insects are less susceptible to pathogens [4], [5], [6], [7], [8], [9]. Fitness benefits conferred by PI may partially explain the prevalence of *Wolbachia* strains that do not confer the more familiarly known reproductive manipulations such as cytoplasmic incompatibility. For example, some *Drosophila* species infected with specific *Wolbachia* strains have greater resistance to viral pathogens compared to their uninfected counterparts [4], [9], [10]. From an applied standpoint, mosquito vectors artificially transinfected with *Wolbachia* exhibit PI against diverse pathogens [5], [6], [8]. The heterologous association between *Wolbachia* and novel host seems to strongly induce this phenotype in mosquitoes, as the native *Wolbachia* strain in many vectors does not generally affect pathogen transmission [6], [8]. *Wolbachia* does cause a small reduction in West Nile virus titer in *Culex quinquefasciatus*, but this effect is subtle and is unlikely to affect the vector competence of the mosquito [7]. In *Aedes aegypti*, artificial *Wolbachia* infections suppress diverse pathogens including RNA viruses, filarial nematodes and the avian malaria parasite *Plasmodium gallinaceum* [5], [6], [8]. In *Anopheles* mosquitoes, somatic infection with the *Wolbachia* strain wMelPop suppresses the rodent malaria parasite *P. berghei*. These results show that *Wolbachia*-induced PI may be of use to control various vector-borne diseases [11].

Although the mechanism behind *Wolbachia*-induced PI is uncertain, several non-mutually exclusive hypotheses have been proposed. In wMelPop and wAlbB-transinfected *Ae. aegypti*, there is induction of the basal immune state of the host by the novel *Wolbachia* strain [5], [6], [8]. Activation of the immune state before the mosquito is challenged with pathogens may make the insect less susceptible to infection. Additionally, there is evidence for resource competition between *Wolbachia* and pathogens such as dengue virus, where virus was only observed in mosquito cells that were not infected with *Wolbachia* [6].

In addition to PI and manipulation of host reproduction, the wMelPop strain of *Wolbachia* causes life shortening in both *Drosophila* and transinfected *Aedes aegypti* [12], [13]. Due to the extrinsic incubation period (EIP) of many pathogens, life shortening can have a dramatic effect on reducing pathogen transmission. As such, wMelPop has been proposed to control vector-borne diseases by skewing the age structure of the mosquito population toward the younger age classes that are not old enough to transmit pathogens [14], [15]. The dual effect of life shortening and PI can act synergistically, enhancing the prospects for *Wolbachia*-based disease control strategies [5], [6], [12].

Although naturally uninfected, *Anopheles* mosquitoes are amenable to *Wolbachia* infection, both *in vitro* [16] and in the mosquito somatic tissues [17]. Somatic infection of insects allows for evaluation of *Wolbachia* phenotypes in the absence of a stably infected host. Recently, somatic infection by wMelPop in *An. gambiae* was shown to reduce *P. berghei* levels in conjunction with induction of several innate immune genes. However, immune up-regulation was only investigated at a single time point [11]. It is unknown whether immune induction occurs constantly throughout the life of the mosquito, whether *Wolbachia* infection will modulate *Plasmodium* species that are important for human health concerns, or whether different *Wolbachia* strains will induce similar phenotypes.

To address these issues, we characterized the infection dynamics of two divergent *Wolbachia* strains (wMelPop and wAlbB) in somatically infected *An. gambiae*, using fluorescence *in situ* hybridization (FISH) and qPCR. Host immune gene expression in response to *Wolbachia* infection was assessed at multiple time points throughout the lifespan of the mosquito. *Wolbachia* mediated PI was evaluated for the human pathogen *P. falciparum*. We show that the mosquito immune response to *Wolbachia* is dynamic, switching between induction and suppression as the mosquito ages. We examined life history traits of mosquitoes infected with the life shortening strain of *Wolbachia* wMelPop, before and after bloodmeals, and show that strong life shortening was only observed immediately after bloodfeeding. The results are discussed in terms of potential applications for using *Wolbachia* as part of a strategy for malaria control.

## Results/Discussion

**Figure 1:**
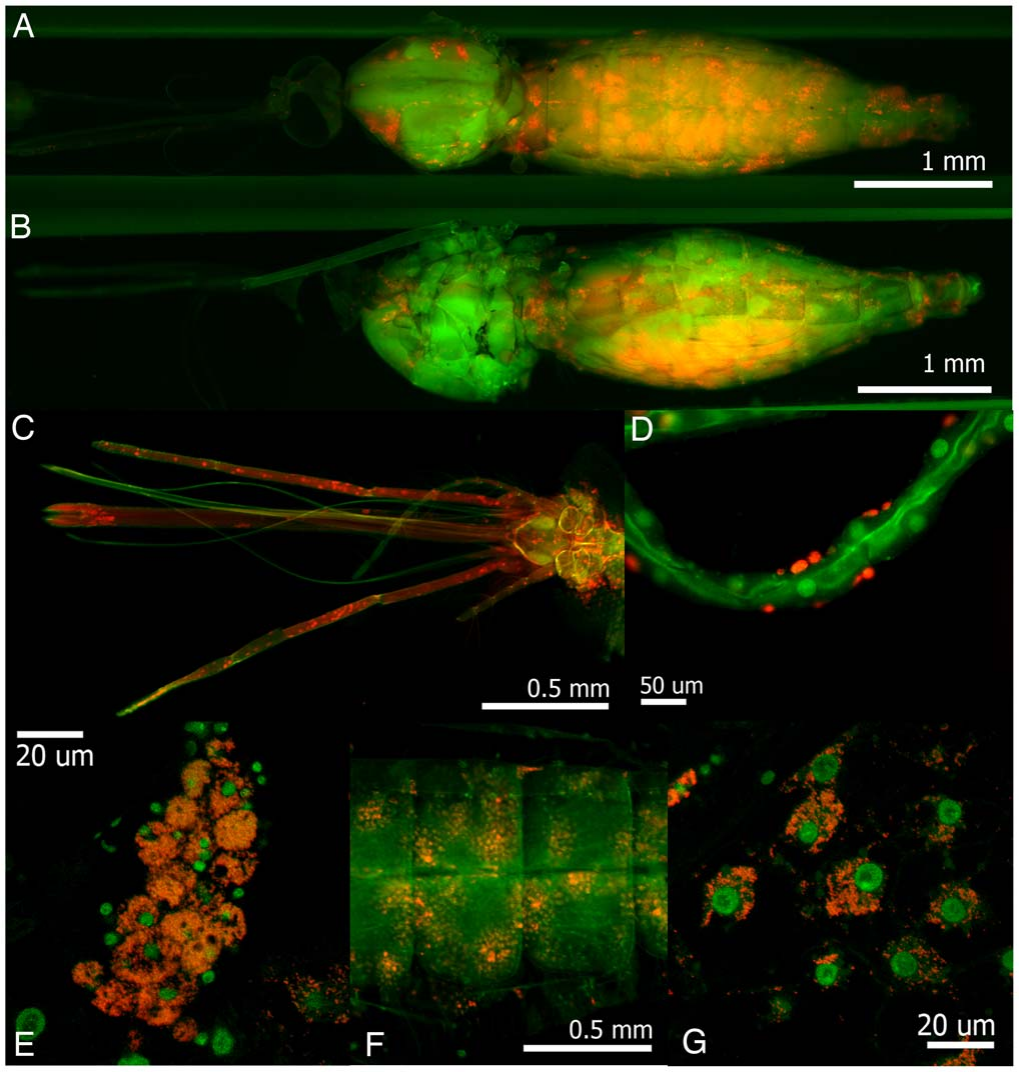
Whole mount fluorescence *in situ* hybridization of *Wolbachia*-infected *An. gambiae*, 30 days post-injection (dpi). *Wolbachia* is distributed throughout the mosquito. (A) Dorsal view of whole mosquito. (B) Lateral view of whole mosquito. (C) *Wolbachia* present in the head, mouthparts and antennae of the mosquito. (D) *Wolbachia* is present in hemocytes adhering to Malpighian tubules. (E) *Wolbachia* infecting the fat body (F) *Wolbachia* present in the abdomen from ventral view. (G) Intracellular *Wolbachia* infecting cells. Scale bars are present for each panel. Red, *Wolbachia*. Green, mosquito cell nuclei. Images with individual green and red channels are presented in Figure S3.

Using whole mosquito fluorescence *in situ* hybridization (FISH), we determined that the *Wolbachia* strain wMelPop disseminates throughout the mosquito and infects numerous tissues after somatic infection by thoracic microinjection. By 30 days post-infection, *Wolbachia* is ubiquitous in the abdomen, where it primarily resides within cells of the fat body, and in cells that adhere to the Malpighian tubules, which are most likely hemocytes that have phagocytized *Wolbachia*. The fat body and hemocytes are major immune tissues within the mosquitoes and infection of these tissues could potentially affect immune processes. Previously it had been demonstrated that *Wolbachia* could replicate within *Anopheles* mosquitoes, however the cellular orientation of the infection was unknown [17]. The occurrence of *Wolbachia* within fat body and hemocyte cells demonstrate conclusively that *Wolbachia* have the capacity to enter, replicate and survive intracellularly in specific somatic tissues within *Anopheles*. This observation is supported by *in vitro* experimentation where *Wolbachia* has established infections in *Anopheles* cell culture [16]. *Wolbachia* are also observed to infect the head of the insect, possibly in the brain or pericerebral fat body. Infection is also observed within the mouthparts and sensory organs of the mosquito (Figure 1) – whether these *Wolbachia* are free in the hemolymph or contained within circulating hemocytes remains to be determined. The distribution of *Wolbachia* in somatically infected *An. gambiae* in part resembles that of the stably infected *Aedes aegypti* [6], [12]. One noticeable difference between the two mosquito species is the lack of infection in the *Anopheles* midgut and germline (Figure S1).

Although adult microinjection has successfully been adapted to transinfect multiple insect species [18], [19], [20], no evidence was found for entry of wMelPop into the *An. gambiae* germline. Previously, adult injection was successfully used to re-infect *D. melanogaster* with wMel, and to establish infection in *Ae. aegypti* with wAlbA and wAlbB [18], [20]. *Laodelphax striatellus,* which naturally harbors wStri, was co-infected with wRi using adult microinjection [19], while wStri has been transferred to *Nilaparvata lugens* by nymphal injection [21]. In *D. melanogaster*, *Wolbachia* was localized to the somatic stem cell niche in the germarium [20], while in both *Ae. aegypti and L. striatellus*, progeny of microinjected females were infected suggesting entry of *Wolbachia* into the germline [18], [19]. In contrast, and similar to our results, somatic infection of *Bombyx mori* was successful after microinjection of *Wolbachia* into immature life stages, but germline infection was not established [22]. Using FISH, no signal was detected in mature ovaries or immature ovarioles in *Anopheles* (Figure S1).

The lack of infection of the *An. gambiae* germline may go some way to explain the unique biology of the *Anopheles* genera, which is naturally uninfected in nature and seems to be impervious to *Wolbachia* transinfection despite numerous attempts. There are many possibilities that may explain the lack of infection in the ovary. While *Wolbachia* can survive intracellularly in *Anopheles* mosquitoes, the ovarian milieu may be inhospitable to the bacteria. Alternatively, ovarian cell receptors that *Wolbachia* utilizes may be too divergent in *Anopheles,* preventing entry into the ovary. Infection itself may cause reproductive ablation. Amhed and Hurd [23] demonstrated that apoptosis in ovarian follicular epithelial cells occurs when the melanization response or humoral antimicrobial activity is induced in *An. gambiae*. Alternatively, constraints to infection may be related to the bacteria. It is evident that *Wolbachia* can adapt to new host backgrounds [24], and certain strains of *Wolbachia* may be more or less suitable for infection establishment. Experiments that address these hypotheses may provide a mechanistic basis for the inability of *Wolbachia* to infect the *Anopheles* germline and may provide clues that could ultimately lead to transinfection of this genus.

**Figure 2:**
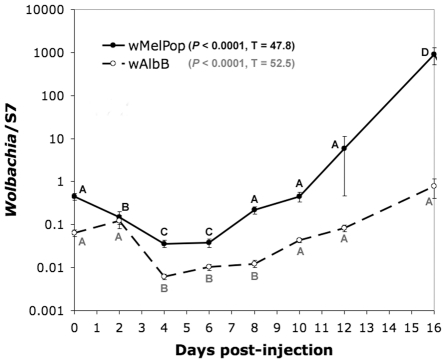
Changes in titer of wMelPop and wAlbB in *An. gambiae* after microinjection, assessed by quantitative PCR. Values are expressed as a ratio of *Wolbachia* genomes to host genomes. Kruskal-Wallis statistics are shown in the legend. *Wolbachia* strains were not statistically compared to one another. Within treatments, time points with the same letter do not differ statistically.

Quantitative PCR (qPCR) analysis demonstrated that *Wolbachia* multiples within the mosquito. Since we do not know whether *Wolbachia* are polyploid, results are presented as *Wolbachia* genomes per host genome. After microinjection, there is an initial decrease in bacterial density before *Wolbachia* replicates to increase in abundance (Figure 2). These results are in concordance with Jin et al [17] who used standard PCR to assess somatic infection dynamics of the wMelpop *Wolbachia* strain. Here, we quantify both wMelpop and wAlbB infection with qPCR and find both these *Wolbachia* strains display a similar infection pattern, although wAlbB densities are several orders of magnitude lower than wMelPop. This is not unexpected as wMelPop, an over replicating strain, replicates faster than wAlbB in the mosquito (Figure 2) and is initially extracted from cell culture and microinjected into the mosquito at higher densities. It is also possible that the ploidy of wMelPop is higher than wAlbB.

**Figure 3:**
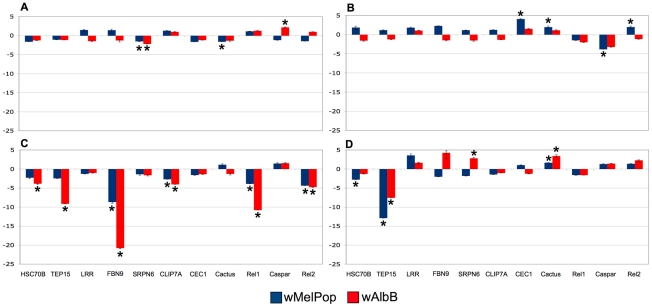
Quantitative rtPCR of immune related genes regulated by *Wolbachia*. Expression was assessed in mosquitoes injected with either wMelPop or wAlbB, compared to a Mos55 cell lysate- injected control. (A) 3 days post-infection (dpi), (B) 6 days dpi, (C) 10 days dpi, (D) 15 days dpi. Blue and red bars represent wMelPop- and wAlbB-infected mosquitoes, respectively. Asterisks denote significantly regulated genes. Error bars represent the maximum and minimum range of expression.

In contrast to *Ae. aegypti* stably infected with *Wolbachia*, we see that the immune response in *Anopheles* after somatic infection is dynamic. At 3 days post infection there is minimal effect on gene expression. Infection by wMelPop and wAlbB moderately suppress Serpin6. wMelPop moderately suppresses cactus, the negative regulator of the Toll pathway, while wAlbB moderately induces Caspar, the negative regulator of the IMD pathway. At 6 days post-infection, Caspar is suppressed by wMelPop in conjunction with up-regulation of Rel2 and cecropin, as well as modestly up-regulating cactus. This time period is coincident with the initial clearing of infection measured by qPCR (Figure 2), and is similar to observations by Kambris and colleagues [11] who observed immune up-regulation (including strong cecropin induction) at a similar time point (8 days post-infection). wAlbB infected mosquitoes display a different profile at this time point, with gene expression not significantly affected. However, at 10 post-infection, the pattern changes to dramatic down-regulation of many immune-related host genes in response to both *Wolbachia* strains, including FBN9, Heat shock 70, CLIP7A, TEP15 and the transcription factors Rel1 and Rel2 (Figure 3). This time period corresponds with *Wolbachia* replication in the mosquito (Figure 2), suggesting that *Wolbachia* may be actively manipulating host gene expression to mediate the infection and replication process. In several instances, suppression of host gene expression by wAlbB is greater compared to wMelPop, suggesting there are strain-specific responses in addition to differences related to bacterial density. This down-regulation is in agreement with regulation patterns observed *in vitro*, where the *Wolbachia* strains wAlbB and wRi suppressed many host genes (including genes associated with innate immunity) in cultured *An. gambiae* Sua5B cells [25]. By 15 days post infection, the response is mixed, with some genes up-regulated and some down-regulated in a *Wolbachia* strain-specific manner (Figure 3).

**Figure 4:**
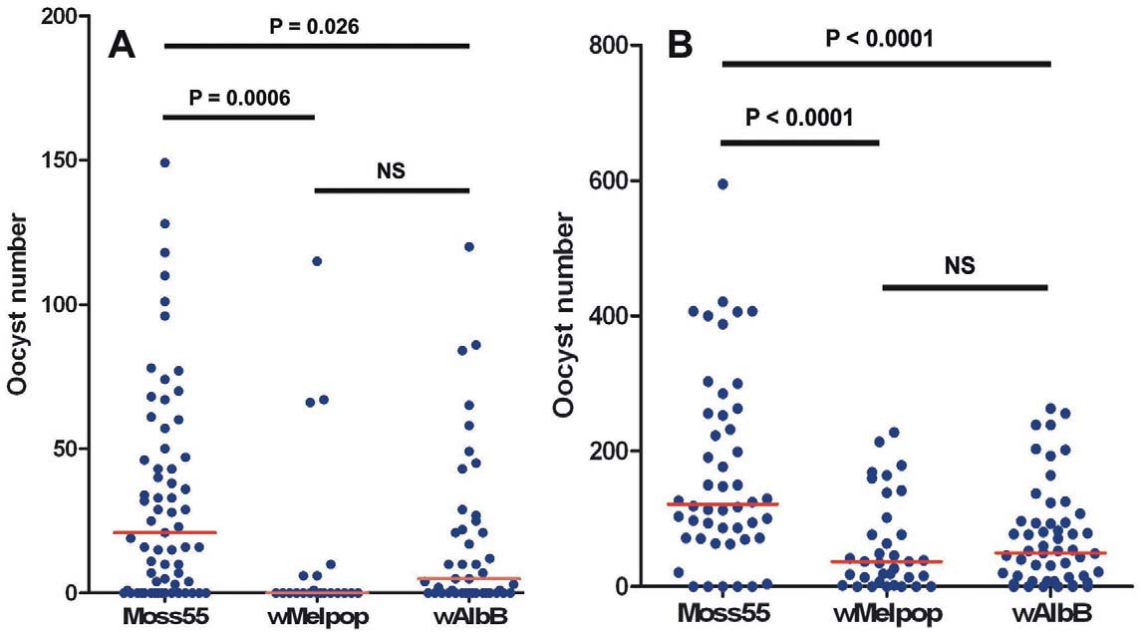
*Plasmodium falciparum* oocyst counts in *Wolbachia*-infected *An*. *gambiae* mosquitoes. Each dot represents a single mosquito. Red lines represent median values. A) low gametocytemic culture; B) high gametocytemic culture. Both wMelPop and wAlbB suppress *P. falciparum* oocyst levels compared to Mos55 cell lysate-injected controls. Infection prevalence (percentage of mosquitoes with one or more oocysts per midgut) was statistically reduced in wMelPop-injected mosquitoes in the low gametocytemic replicate (see text).

After somatic infection, *P. falciparum* oocyst development was significantly reduced (40–60%) by both wMelPop and wAlbB compared to the Mos55 (*Anopheles* cell extract) injected control. We observed similar results using both low gametocytemic and high gametocytemic *Plasmodium* cultures (Figure 4). In the low gametocytemic replicate, infection prevalence (percentage of mosquitoes with one or more oocysts per midgut) was statistically reduced in wMelPop-injected mosquitoes (Mos55: 75%, N = 65; wMelPop: 33%, N = 21; wAlbB: 60%, N = 45; d.f. = 2, Cramer's V = 0.39, *P* = 0.002). Infection prevalence did not differ statistically in the high gametocytemic replicates (Mos55: 90%, N = 50; wMelPop: 83%, N = 35; wAlbB: 84%, N = 55). No correlation was observed between *Wolbachia* density and *Plasmodium* oocyst load for either *Wolbachia* strain (Figure S2), suggesting that the reduction of *Plasmodium* is not directly related to *Wolbachia* density (i.e. mosquitoes with high oocyst levels did not necessarily have the lowest *Wolbachia* titers).

While wMelPop moderately induces the mosquito immune system at 6 days post-infection, by 10 days post-injection, the majority of tested immune genes were down-regulated by both *Wolbachia* strains (Figure 3). These time points correlate to when *Plasmodium* is developing within the mosquito midgut. Although, Kambris et al [11] provide evidence that wMelPop-mediated immune up-regulation induces PI in *Anopheles* against *P. berghei*, our data suggest that the mosquito immune response to *Wolbachia* is more dynamic. The modulation of the later immune response suggests mechanisms other than stimulation of basal immunity may be involved in PI in *An. gambiae*. Alternatively, immune up-regulation around the initial infection period when ookinetes are invading the midgut may be sufficient for a decrease in *Plasmodium* load. Possibly these different mechanisms may be acting in concert. A more thorough analysis of global immune regulation in response to *Wolbachia* infection throughout the life of the insect may clarify this issue.

In our *Plasmodium* experiments, we noted higher mortality of wMelPop-injected mosquitoes compared to wAlbB or cell homogenate-injected treatments. Our previous data suggested that somatic infections of wMelPop were not virulent to *Anopheles gambiae* [17]. However, in those experiments mosquitoes were not allowed access to blood. We therefore considered the hypothesis that wMelPop-induced virulence in *Anopheles gambiae* was conditional on bloodfeeding.

**Figure 5:**
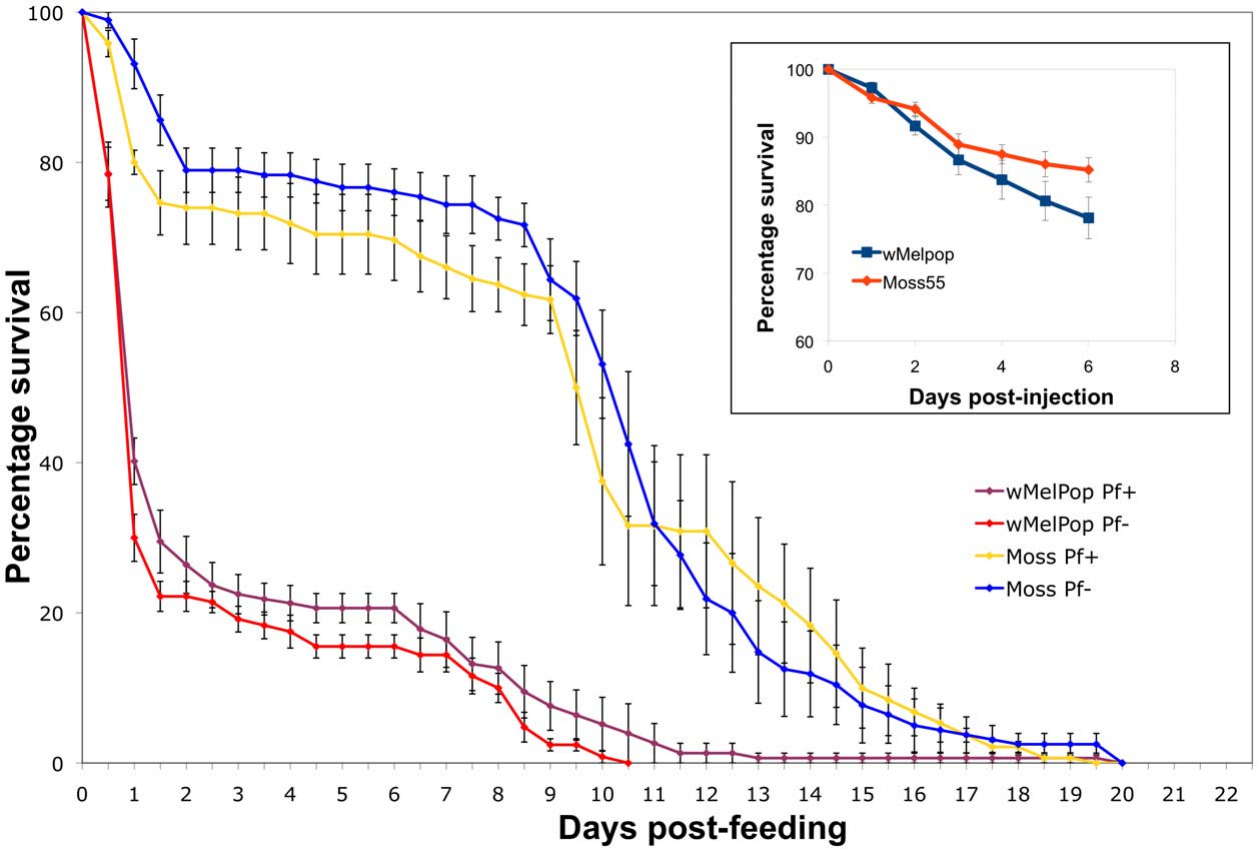
Mortality of wMelPop-infected *An*. *gambiae* mosquitoes. Inset: Mortality of wMelPop or Mos55 cell lysate-injected mosquitoes prior to bloodfeeding. Large graph: Mortality of wMelPop or Mos55 cell lysate-injected mosquitoes after feeding on *P*. *falciparum* infected or uninfected human blood. Mosquitoes were collected every 24 hours pre-bloodmeal or every 12 hours post-bloodmeal. After bloodfeeding, there is a dramatic increase in mortality of wMelPop-infected mosquitoes, resulting in approximately 80% mortality after 3 days post-feeding. The slope of the mortality trajectory of surviving mosquitoes is similar to controls. wMelPop treatments differ from Mos55 treatments (*P*<0.0001), but *Plasmodium* infection status was not significant.

Mosquitoes were injected with wMelPop or with uninfected cell culture homogenate as previously described, held for 7 days, then were offered a human bloodmeal with or without *P. falciparum* parasites through a membrane feeder. After bloodfeeding, fed mosquitoes were separated from unfed mosquitoes and their mortality trajectories assessed. We observed that prior to bloodfeeding, there were no dramatic differences in mortality between infected and uninfected mosquitoes, similar to previous observation. However, wMelPop-infected mosquitoes exhibited a dramatic increase in mortality between 12–24 h post-bloodmeal. After 3 days approximately 80% of the mosquitoes died. After this period, the mortality trajectories of the two treatments become similar again (Figure 5). Infection with *Plasmodium* made no difference in the mortality phenotypes. Interestingly, we also noted that when comparing *Wolbachia* levels to *Plasmodium* oocyst levels, *Wolbachia* titers were much lower in assayed wMelPop-infected mosquitoes compared to wAlbB mosquitoes (Figure S2), suggesting that mosquitoes with high wMelPop titers did not survive long enough to be assayed for *Plasmodium* infection. These data show that wMelPop is virulent to *An. gambiae*, but the virulence phenotype is different than that described for *Ae. aegypti* and *Drosophila* [12], [13]. Instead of a general increase in lifetime mortality rates, we observe an acute increase in mortality directly related to bloodmeal acquisition and/or digestion.

Post bloodmeal, multiple developmental and metabolic processes occur which drastically alter mosquito physiology. Alteration of any of these processes by *Wolbachia* may potentially induce mortality. In cultured *Anopheles* Sua5B cells, *Wolbachia* infection down-regulates host expression of multiple antioxidant genes, including peroxiredoxin, superoxide dismutase and glutathione S transferase [25]. In bloodfed mosquitoes, antioxidant transcripts are up-regulated post bloodmeal [26], [27], [28], [29], [30]. A blood meal also increases iron levels, which are a precursor to reactive oxygen species (ROS). In other systems, *Wolbachia* has been seen to influence the expression of ferritin and plays a role in iron metabolism [31], [32]. We hypothesize that modulated levels of ROS within the mosquito may be the cause of post bloodmeal mortality. Lending credence to this hypothesis is the observation of increased mortality post-bloodmeal in *An. gambiae* after silencing of anti-oxidant genes [33]. The more striking mortality observed in this study may be due to down-regulation of numerous genes. Additionally, blood feeding is known to spark a proliferation of bacteria within the insect [1]. In *Ae. aegypti,* the expansion of gut bacteria post blood meal is attributed to a reduction in ROS, which can result in death of the mosquito [34]. Here, pathogenicity may be directly linked to wMelPop levels or indirectly by *Wolbachia* influencing the density of other bacteria. Alternatively, the effect of wMelPop on other physiological processes that occur after a blood meal (such as vitellogenesis or nutrient metabolism) may cause fitness costs, as seen in *Ae. aegypti* where wMelPop affects reproductive output when mosquitoes were fed on non-human hosts [35]. If stable *Anopheles* infections behave in a similar manner to somatic infections, this acute mortality phenotype could inhibit CI-induced drive of wMelPop into mosquito populations, and provide a selection pressure against the life-shortening phenotype as a large proportion of mosquitoes may die before producing offspring. These potential pitfalls could be offset by the use of this phenotype in a population suppression strategy, or the use of non-virulent *Wolbachia* strains such as wAlbB.

The use of *Wolbachia* to control arthropod-borne disease has been postulated for some time. Previous ideas centered on the use of *Wolbachia* as a gene drive agent, however now it is evident that *Wolbachia* can also inhibit pathogen development in insects [4], [5], [6], [7], [8], [9]. The obvious limitation to this approach for malaria control is the failure to create a *Wolbachia* infected *Anopheles* line, and this still remains a massive challenge in the field of *Wolbachia* biology. Here we have shown that *An*. *gambiae* mosquitoes somatically infected by two strains (wMelPop or wAlbB) are less susceptible to the major human malaria parasite *P. falciparum.* Using FISH and qPCR, *we* determined that *Wolbachia* has ubiquitous distribution in many mosquito tissues and replicates within the *Anopheles* host. As one oocyst is capable of producing many sporozoites, it would be interesting to determine if sporozoite number is reduced by *Wolbachia* considering the vast tissue distribution in somatically infected mosquitoes. The results suggest that *An. gambiae* stably infected with *Wolbachia* may have reduced ability to maintain transmission of *Plasmodium* by multiple strain-dependent mechanisms.

## Materials and Methods

### Ethics statement

Anonymous expired human blood was obtained from a local blood bank for use in mosquito blood feeding experiments.

### *Wolbachia* culture and mosquito infection

*Wolbachia* was cultured and extracted from infected *Anopheles* cells as previously described [16], [36]. *An. gambiae* mosquitoes (Keele strain) were reared as described [16]. Two days post emergence, adult female mosquitoes were anesthetized on ice and injected with *Wolbachia* according to previously established methodology [17]. Post injection, mosquitoes were incubated at 19°C for 2 days for recovery then maintained at 28°C.

### Fluorescence *in situ* hybridization (FISH)

FISH was performed on wMelPop infected mosquitoes 30 days post injection following the experimental procedure outlined by Koga et al. [37]. Briefly, mosquitoes were fixed in acetone for 3 months, legs were removed and mosquitoes were secondarily fixed in Carnoy's solution. To minimize autofluorescence, mosquitoes were transferred to 10% hydrogen peroxide in 6% alcohol for 5 days. After rehydration in PBST (1–2 hours), tissues were pre-hybridized followed by hybridization with the *Wolbachia* specific probe overnight [38]. Samples were washed in PBST 3 times to remove excess probe, counterstained with SYTOX green (Invitrogen) and visualized by epifluorescent and confocal microscopy. Individual channel images are available as Supplementary data (Figure S3). FISH controls included 1) no probe controls, 2) competition controls in which unlabeled oligonucleotides were added to the hybridization buffer to suppress the fluorescent signals and 3) RNase digestion controls, in which prior to hybridization RNAs in the insect materials were removed by RNase A treatment (Figure S4).

### Quantitative PCR (qPCR) for *Wolbachia* density and host gene expression

DNA or RNA was extracted from somatically infected mosquitoes using DNAzol (Molecular Research Center, Inc., Cincinnati, OH) or RNeasy mini kits (Qiagen) for estimation of *Wolbachia* density and quantification of host gene expression respectively. qPCR to determine the density of wMelPop in whole mosquitoes was completed by amplifying the single copy gene WD_0550 [24], while wAlbB was amplified with modified GF and BR primers which specifically bind to the wsp gene [18]. Ten mosquitoes were assay at each time point for each strain to estimate *Wolbachia* density, while 5 mosquitoes were used for host gene expression per time point. The relative abundance of each *Wolbachia* strain was determined after normalization to the mosquito single-copy S7 gene [39]. For host gene expression, RNA was DNase treated (Ambion) and cDNA synthesized using superscript III (Invitrogen) following manufactures guidelines. qPCR was completed using a Rotor gene Q (Qiagen) using the Rotor gene SYBR green PCR kit (Qiagen) according to manufactures guidelines. qPCRs were completed in triplicate. PCR primers are listed in Table S1. Melt curve analysis was completed on all PCRs. In *Wolbachia* density experiments, data were analyzed by Kruskal-Wallis test using the Connover-Inman method for pairwise contrasts between time points. For host gene expression experiments, significance was assessed by Mann-Whitney U test compared to mosquitoes injected with uninfected Mos55 cell culture homogenate (control). Tested mosquito genes were identified in a microarray screen of *Wolbachia*-regulated *Anopheles* genes in cultured cells [25]. Additional analyses were conducted using REST [40] and qGENE [41] software.

### *Plasmodium falciparum* mosquito infections

2-day old female mosquitoes were intrathoracically injected with wMelPop or wAlbB (purified from cell culture) as described [17] or with uninfected Mos55 cell culture homogenate (control). Seven days post-injection, mosquitoes were offered a *Plasmodium*-infected blood meal. Prior to blood feeding, mosquitoes were starved overnight. The gametocytemia of infected blood meals was approximately 0.3% and 1% for low and high titer infections, respectively. After blood feeding, unfed mosquitoes were removed. *P. falciparum* NF-54 gametocyte cultures were washed and mosquitoes were fed infected blood warmed to 37°C through a membrane feeder [42]. Post feeding, unfed mosquitoes were removed and blood-fed *An. gambiae* were incubated at 24°C for 7 days. Midguts of mosquitoes were dissected, stained with 0.2% mercurochrome and oocysts enumerated using a light contrast microscope (Olympus). The *Wolbachia* density of each mosquito carcass was determined by qPCR as described above. The experiment was replicated 3 times. Replicate one was a high-gametocytemic culture, while replicates two and three had low gametocytemia. The variances of the data for replicates two and three did not differ statistically and were pooled for analysis (squared ranks test, *P*>0.05) while replicate one was analyzed separately. Data were analyzed by Kruskal-Wallis test using the Dwass method for pairwise comparisons.

### wMelPop mortality experiments

*An. gambiae* female adults were injected with wMelPop or uninfected Mos55 cell culture homogenate (control) and fed a *P. falciparum* gametocyte infected or uninfected blood meal as previously described. Unfed mosquitoes were separated from fed mosquitoes. Mosquitoes were reared at 24°C at a density of approximately 30 mosquitoes per cup (4 cups per treatment) and monitored twice daily for survival. Dead mosquitoes were removed from the experiment every 12 hours. The entire experiment was repeated twice. Data were analyzed by Kaplan-Meier analysis. Statistical significance was assessed by Kruskal-Wallis test using the Dwass method for pairwise comparisons.

## Supporting Information

Figure S1 FISH of wMelPop somatically-infected *Anopheles gambiae* tissues. (A) midgut. (B) immature ovarioles. (C) Mature eggs. *Wolbachia* is not observed in midgut or ovaries.
(DOC)

Figure S2 Lack of correlation between *Wolbachia* levels in the mosquito carcass and *Plasmodium falciparum* oocyst levels in the mosquito midgut.
(DOC)

Figure S3 FISH of wMelPop somatically-infected *Anopheles gambiae* tissues with individual red and green channels and overlay image. (A-C) Dorsal view of whole mosquito; (A) red and green channel, (B) green channel only, (C) red channel only. (D-F) Mouthparts and antennae of the mosquito; (D) red and green channel, (E) green channel only, (F) red channel only. (G-I) fat bodies; (G) red and green channel, (H) green channel only, (I) red channel only. (J-L) hemocytes adhering to Malpighian tubules; (J) red and green channel, (K) green channel only, (L) red channel only.
(DOC)

Figure S4 FISH controls (as described in text). A) no probe control; B) competition control; C) RNase control.
(DOC)

Table S1 PCR primers used in this study.
(DOC)

## References

[1] Dong Y, Manfredini F, Dimopoulos G (2009). Implication of the mosquito midgut microbiota in the defense against malaria parasites.. PLoS Pathog.

[2] Werren JH, Baldo L, Clark ME (2008). *Wolbachia*: master manipulators of invertebrate biology.. Nat Rev Microbiol.

[3] Stouthamer R, Breeuwer JA, Hurst GD (1999). *Wolbachia pipientis*: microbial manipulator of arthropod reproduction.. Annu Rev Microbiol.

[4] Hedges LM, Brownlie JC, O'Neill SL, Johnson KN (2008). *Wolbachia* and virus protection in insects.. Science.

[5] Kambris Z, Cook PE, Phuc HK, Sinkins SP (2009). Immune activation by life-shortening *Wolbachia* and reduced filarial competence in mosquitoes.. Science.

[6] Moreira LA, Iturbe-Ormaetxe I, Jeffery JA, Lu G, Pyke AT (2009). A *Wolbachia* symbiont in *Aedes aegypti* limits infection with *Dengue*, *Chikungunya*, and *Plasmodium*.. Cell.

[7] Glaser RL, Meola MA (2010). The native *Wolbachia* endosymbionts of *Drosophila melanogaster* and *Culex quinquefasciatus* increase host resistance to West Nile virus infection.. PLoS ONE.

[8] Bian G, Xu Y, Lu P, Xie Y, Xi Z (2010). The endosymbiotic bacterium *Wolbachia* induces resistance to *Dengue virus* in *Aedes aegypti*.. PLos Pathog.

[9] Teixeira L, Ferreira A, Ashburner M (2008). The bacterial symbiont Wolbachia induces resistance to RNA viral infections in Drosophila melanogaster.. PLoS Biol.

[10] Osborne SE, Leong YS, O'Neill SL, Johnson KN (2009). Variation in antiviral protection mediated by different *Wolbachia* strains in *Drosophila simulans*.. PLoS Pathog.

[11] Kambris Z, Blagborough AM, Pinto SB, Blagrove MSC, Godfray HCJ (2010). *Wolbachia* stimulates immune gene expression and inhibits *Plasmodium* development in *Anopheles gambiae*.. PLoS Pathog.

[12] McMeniman CJ, Lane AM, Cass BN, Fong AWC, Sidhu M (2009). Stable introduction of a life-shortening *Wolbachia* infection into the mosquito *Aedes aegypti*.. Science.

[13] Min KT, Benzer S (1997). *Wolbachia*, normally a symbiont of *Drosophila*, can be virulent, causing degeneration and early death.. Proc Natl Acad Sci USA.

[14] Cook P, McMeniman CJ, O'Neill SL (2008). Modifying insect population age structure to control vector-borne disease.. Adv Exp Med Biol.

[15] Sinkins SP, O'Neill SL, Handler AF, James AA (2000). *Wolbachia* as a vehicle to modify insect populations.. Insect Transgenesis: Methods and Applications.

[16] Rasgon JL, Ren X, Petridis M (2006). Can *Anopheles gambiae* be infected with *Wolbachia pipientis*? Insights from an *in vitro* system.. Appl Environ Microbiol.

[17] Jin C, Ren X, Rasgon JL (2009). The virulent *Wolbachia* strain wMelPop efficiently establishes somatic infections in the malaria vector *Anopheles gambiae*.. Appl Environ Microbiol.

[18] Ruang-Areerate T, Kittayapong P (2006). *Wolbachia* transinfection in *Aedes aegypti*: A potential gene driver of dengue vectors.. Proc Natl Acad Sci USA.

[19] Kang L, Ma X, Cai L, Liao S, Sun L (2003). Superinfection of *Laodelphax striatellus* with *Wolbachia* from *Drosophila simulans*.. Heredity.

[20] Frydman HM, Li JM, Robson DN, Wieschaus E (2006). Somatic stem cell niche tropism in *Wolbachia*.. Nature.

[21] Kawai S, Matsumoto Y, Gotoh T, Noda H (2009). Transinfection of *Wolbachia* in planthoppers: nymphal injection of cultured *Wolbachia* and infection dynamics.. Environ Entomol.

[22] Kageyama D, Narita S, Noda H (2008). Transfection of feminizing *Wolbachia* endosymbionts of the butterfly, *Eurema hecabe*, into the cell culture and various immature stages of the silkmoth, *Bombyx mori*.. Microb Ecol.

[23] Ahmed AM, Hurd H (2006). Immune stimulation and malaria infection impose reproductive costs in *Anopheles gambiae* via follicular apoptosis.. Microbes Infect.

[24] McMeniman CJ, Lane AM, Fong AWC, Voronin DA, Iturbe-Ormaetxe I (2008). Host adaptation of a *Wolbachia* strain after long-term serial passage in mosquito cell lines.. Appl Environ Microbiol.

[25] Hughes GL, Ren X, Ramirez JL, Sakamoto JM, Bailey JA (2011). Wolbachia infections in *Anopheles gambiae* cells: transcriptomic characterization of a novel host-symbiont interaction.. PLoS Pathog.

[26] Sanders HR, Evans AM, Ross LS, Gill SS (2003). Blood meal induces global changes in midgut gene expression in the disease vector, *Aedes aegypti*.. Insect Biochem Mol Biol.

[27] Ribeiro JM (2003). A catalogue of *Anopheles gambiae* transcripts significantly more or less expressed following a blood meal.. Insect Biochem Mol Biol.

[28] Marinotti O, Nguyen QK, Calvo E, James AA, Ribeiro JM (2005). Microarray analysis of genes showing variable expression following a blood meal in *Anopheles gambiae*.. Insect Mol Biol.

[29] Holt RA (2002). The genome sequence of the malaria mosquito *Anopheles gambiae*.. Science.

[30] Dana AN, Hong YS, Kern MK, Hillenmeyer ME, Harker BW (2005). Gene expression patterns associated with blood-feeding in the malaria mosquito *Anopheles gambiae*.. BMC Genomics.

[31] Brownlie JC, Cass BN, Riegler M, Witsenburg JJ, Iturbe-Ormaetxe I (2009). Evidence for metabolic provisioning by a common invertebrate endosymbiont, *Wolbachia pipientis*, during periods of nutritional stress.. PLoS Pathog.

[32] Kremer N, Voronin DA, Charif D, Mavingui P, Mollereau B (2009). *Wolbachia* interferes with ferritin expression and iron metabolism in insects.. PLoS Pathog.

[33] Magalhaes T, Brackney DE, Beier JC, Foy BD (2008). Silencing an *Anopheles gambiae* catalase and sulfhydryl oxidase increases mosquito mortality after a blood meal.. Arch Insect Biochem Physiol.

[34] Oliveira JHM, Gonçalves RLS, Lara FA, Dias FA, Gandara ACP (2011). Blood Meal-Derived Heme Decreases ROS Levels in the Midgut of Aedes aegypti and Allows Proliferation of Intestinal Microbiota.. PLoS Pathog.

[35] McMeniman CJ, Hughes GL, O'Neill SL (2011). A *Wolbachia* symbiont in *Aedes aegypti* disrupts mosquito egg development to a greater extent when mosquitoes feed on nonhuman versus human blood.. J Med Entomol.

[36] Rasgon JL, Gamston C, Ren XX (2006). Survival of *Wolbachia pipientis* in cell-free medium.. Appl Environ Microbiol.

[37] Kono M, Koga R, Shimada M, Fukatsu T (2008). Infection dynamics of coexisting beta- and gammaproteobacteria in the nested endosymbiotic system of mealybugs.. Appl Environ Microbiol.

[38] Heddi A, Grenier AM, Khatchadourian C, Charles H, Nardon P (1999). Four intracellular genomes direct weevil biology: nuclear, mitochondrial, principal endosymbiont, and *Wolbachia*.. Proc Natl Acad Sci USA.

[39] Das S, Radtke A, Choi Y-J, Mendes AM, Valenzuela JG (2010). Transcriptomic and functional analysis of the *Anopheles gambiae* salivary gland in relation to blood feeding.. BMC Genomics.

[40] Pfaffl MW, Horgan GW, Dempfle L (2002). Relative expression software tool (REST) for group-wise comparison and statistical analysis of relative expression results in real-time PCR.. Nucleic Acids Res.

[41] Joehanes R, Nelson JC (2008). QGene 4.0, an extensible Java QTL-analysis platform.. Bioinformatics.

[42] Carter R, Ranford-Cartwright L, Alano P (1993). The culture and preparation of gametocytes of *Plasmodium falciparum* for immunochemical, molecular, and mosquito infectivity studies.. Methods Mol Biol.

