# Supplementary material for: Wolbachia Infections Are Virulent and Inhibit the Human Malaria Parasite Plasmodium Falciparum in Anopheles Gambiae
Source: PLoS Pathog. 2011 May 19;7(5):e1002043. doi: 10.1371/journal.ppat.1002043 (PMC3098226; doi:10.1371/journal.ppat.1002043)
Supplement: Figure S1 — FISH of wMelPop somatically-infected Anopheles gambiae tissues. (A) midgut. (B) immature ovarioles. (C) Mature eggs. Wolbachia is not observed in midgut or ovaries. (DOC) [file ppat.1002043.s001.doc]

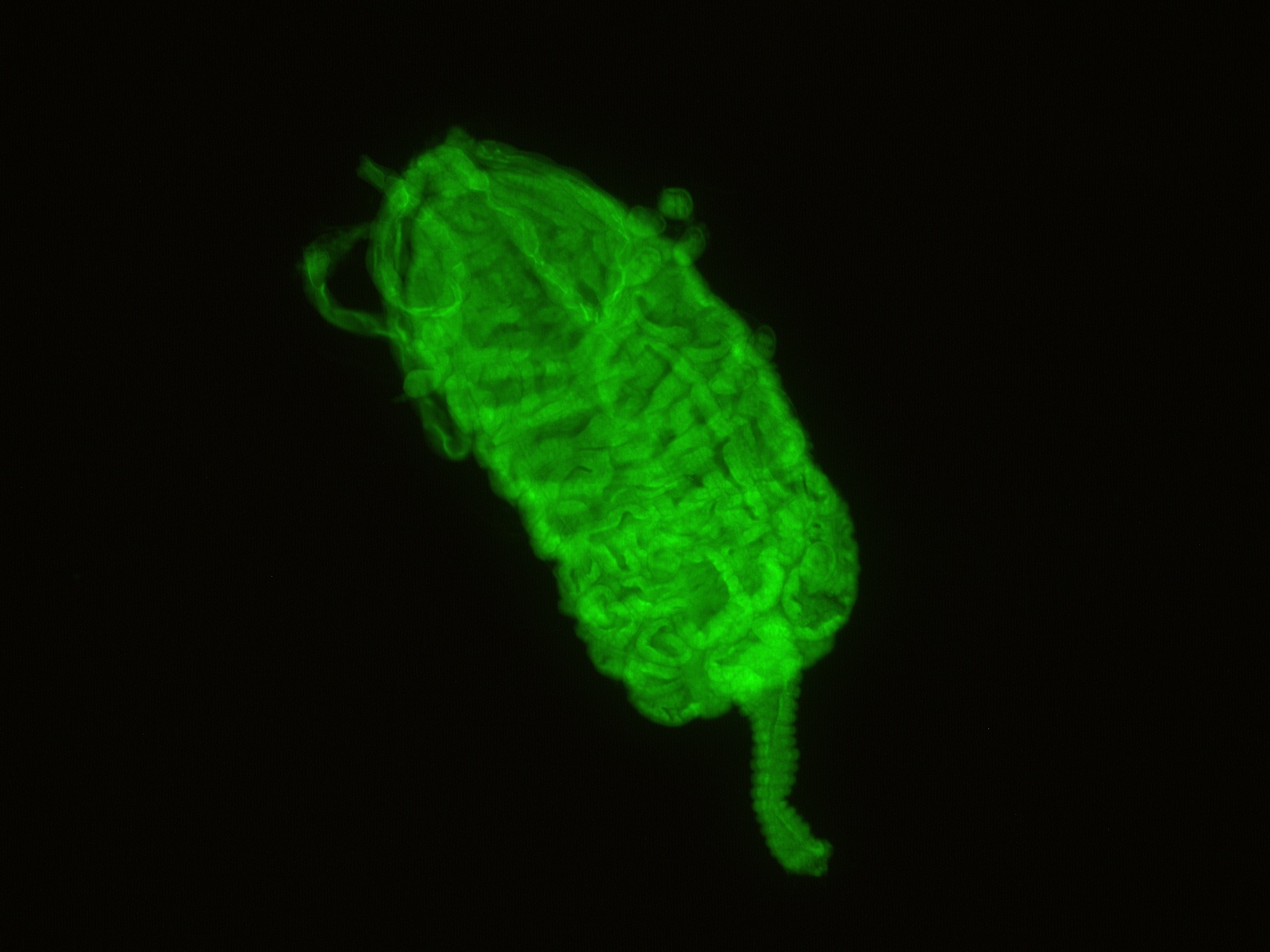

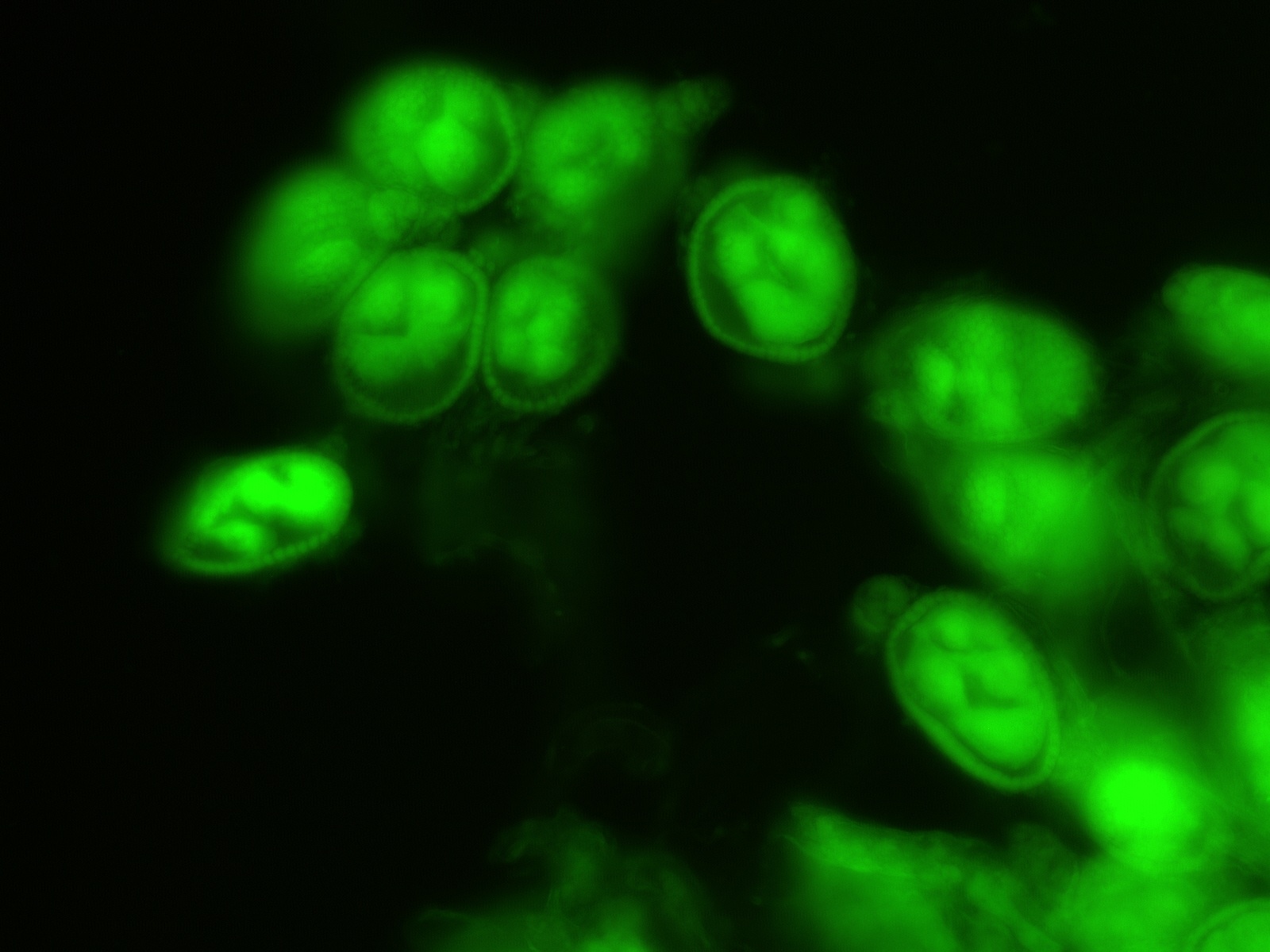

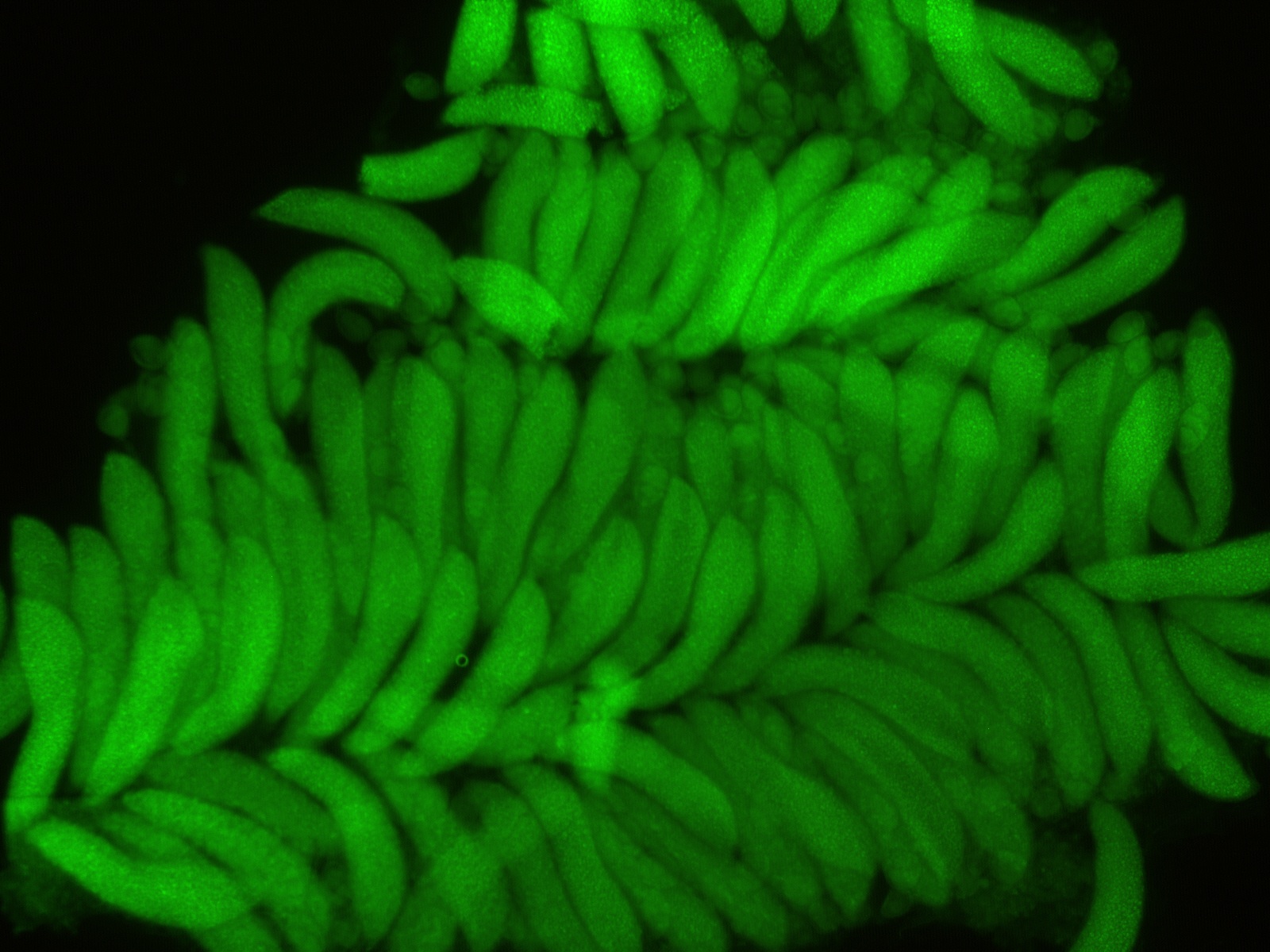


**A**

**B**

**C**

Supplementary Figure 1. FISH of wMelPop somatically-infected *Anopheles gambiae* tissues. (A) midgut. (B) immature ovarioles. (C) Mature eggs. Wolbachia does not infect midgut or ovaries.
