# Supplementary material for: Wolbachia Infections Are Virulent and Inhibit the Human Malaria Parasite Plasmodium Falciparum in Anopheles Gambiae
Source: PLoS Pathog. 2011 May 19;7(5):e1002043. doi: 10.1371/journal.ppat.1002043 (PMC3098226; doi:10.1371/journal.ppat.1002043)
Supplement: Figure S2 — Lack of correlation between Wolbachia levels in the mosquito carcass and Plasmodium falciparum oocyst levels in the mosquito midgut. (DOC) [file ppat.1002043.s002.doc]

**A**

Supplementary Figure 2. Lack of correlation between *Wolbachia* levels in the mosquito carcass and *Plasmodium falciparum* oocyst levels in the mosquito midgut.
