# Supplementary material for: Wolbachia Infections Are Virulent and Inhibit the Human Malaria Parasite Plasmodium Falciparum in Anopheles Gambiae
Source: PLoS Pathog. 2011 May 19;7(5):e1002043. doi: 10.1371/journal.ppat.1002043 (PMC3098226; doi:10.1371/journal.ppat.1002043)
Supplement: Figure S3 — FISH of wMelPop somatically-infected Anopheles gambiae tissues with individual red and green channels and overlay image. (A-C) Dorsal view of whole mosquito; (A) red and green channel, (B) green channel only, (C) red channel only. (D-F) Mouthparts and antennae of the mosquito; (D) red and green channel, (E) green channel only, (F) red channel only. (G-I) fat bodies; (G) red and green channel, (H) green channel only, (I) red channel only. (J-L) hemocytes adhering to Malpighian tubules; (J) red and green channel, (K) green channel only, (L) red channel only. (DOC) [file ppat.1002043.s003.doc]

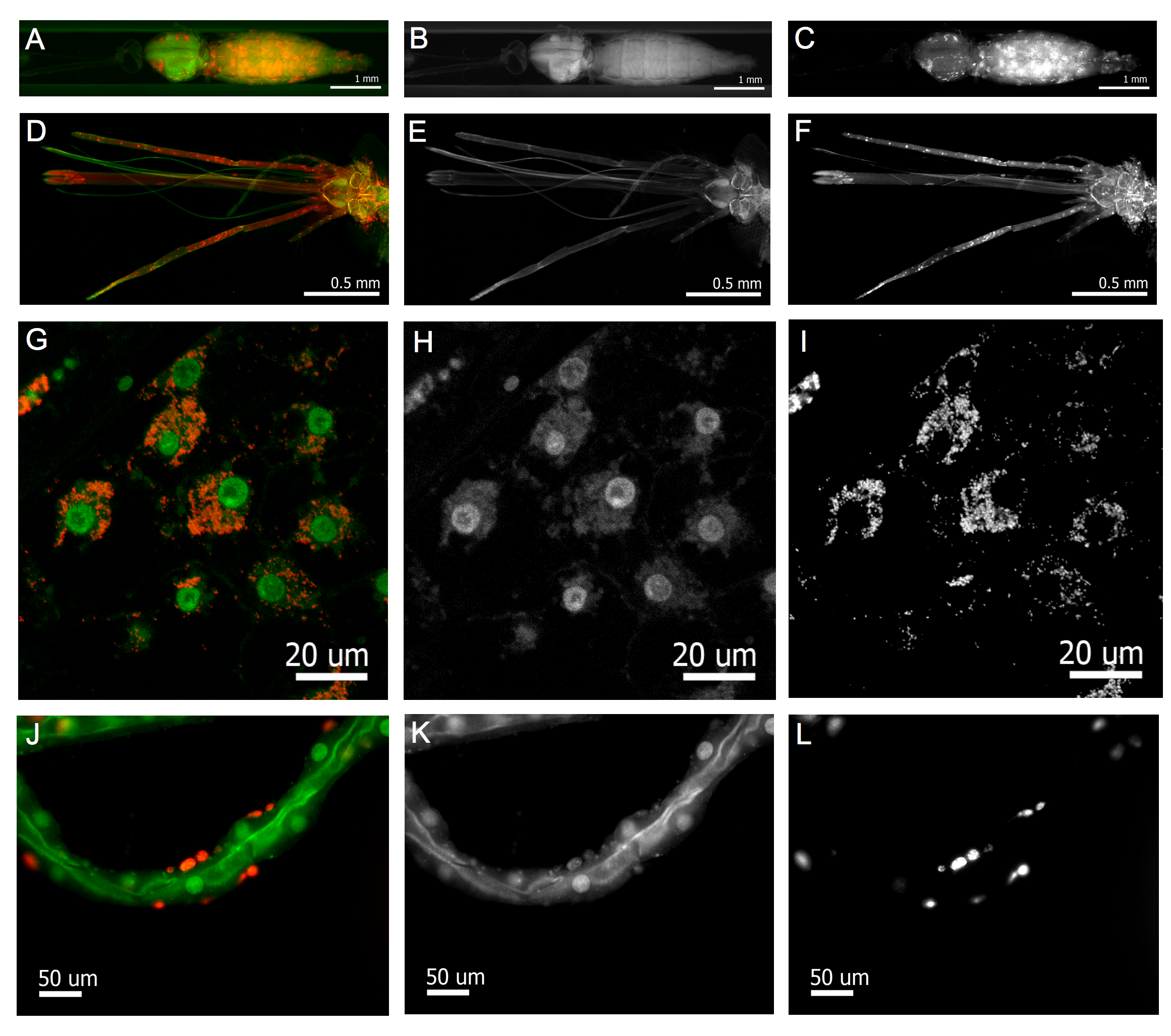


Supplementary Figure 3. FISH of wMelPop somatically-infected *Anopheles gambiae* tissues with individual red and green channels and overlay image. (A-C) Dorsal view of whole mosquito; (A) red and green channel, (B) green channel only, (C) red channel only. (D-F) Mouthparts and antennae of the mosquito; (D) red and green channel, (E) green channel only, (F) red channel only. (G-I) fat bodies; (G) red and green channel, (H) green channel only, (I) red channel only. (J-L) hemocytes adhering to Malpighian tubules; (J) red and green channel, (K) green channel only, (L) red channel only.
