# Supplementary material for: Wolbachia Infections Are Virulent and Inhibit the Human Malaria Parasite Plasmodium Falciparum in Anopheles Gambiae
Source: PLoS Pathog. 2011 May 19;7(5):e1002043. doi: 10.1371/journal.ppat.1002043 (PMC3098226; doi:10.1371/journal.ppat.1002043)
Supplement: Figure S4 — FISH controls (as described in text). A) no probe control; B) competition control; C) RNase control. (DOC) [file ppat.1002043.s004.doc]

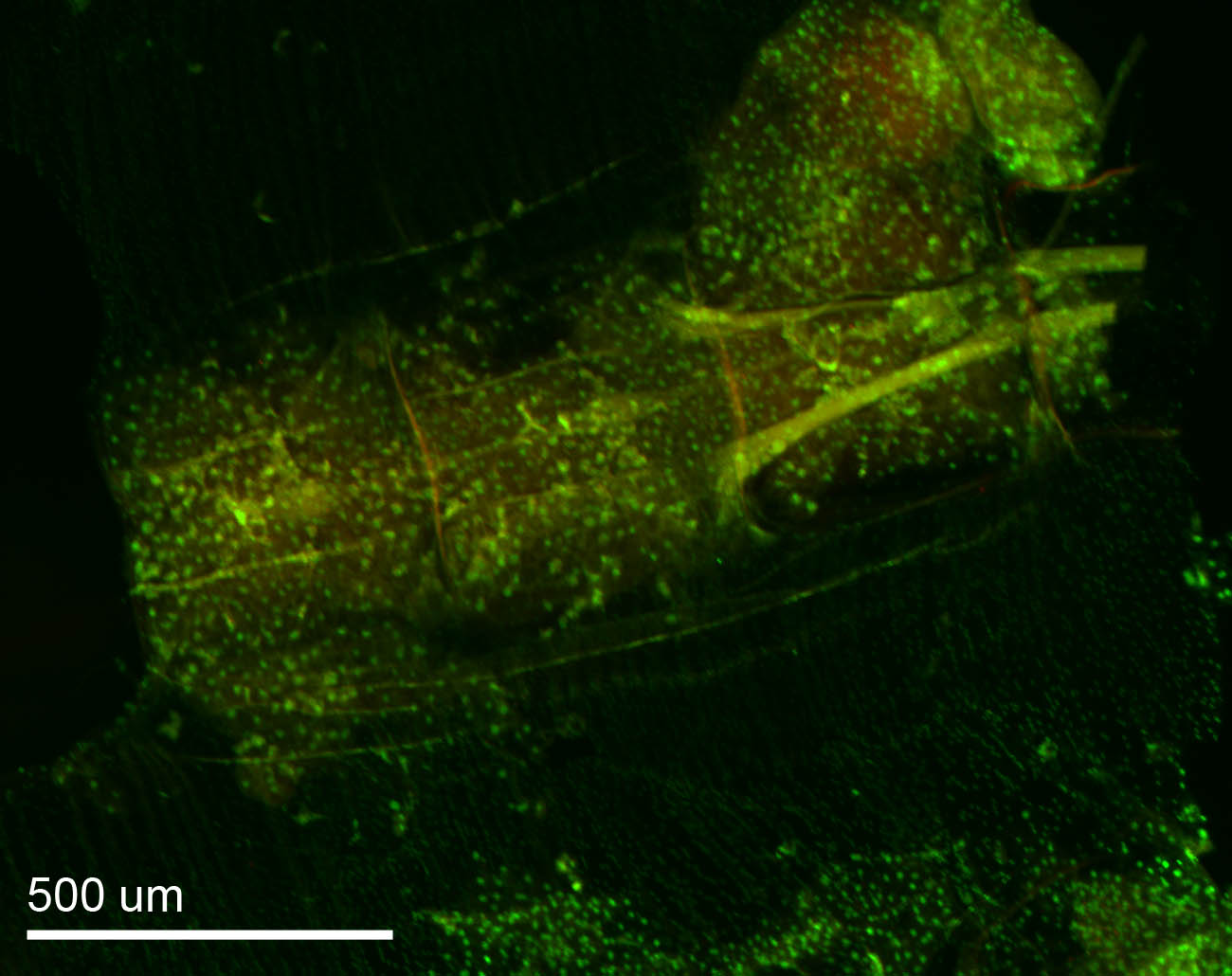

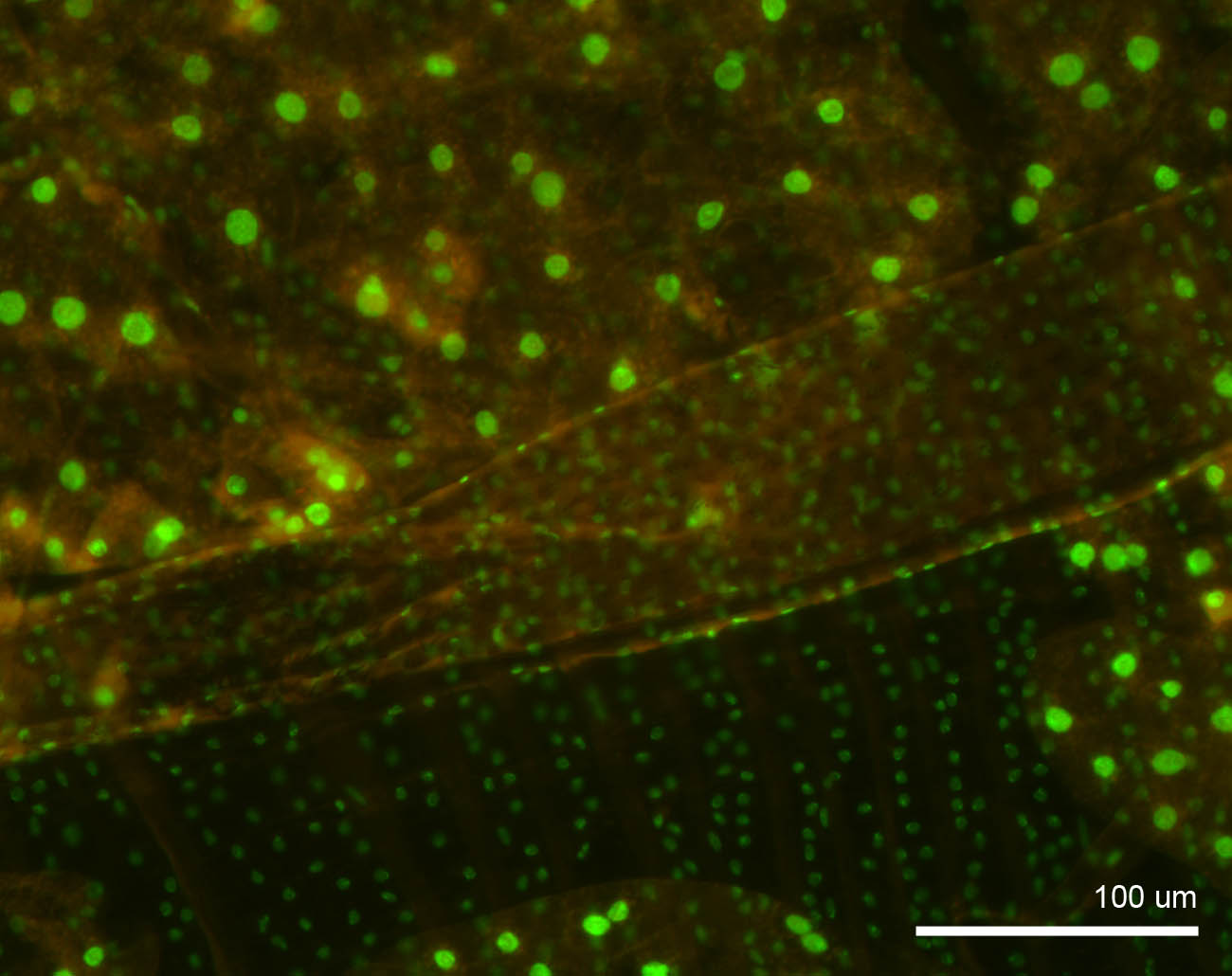

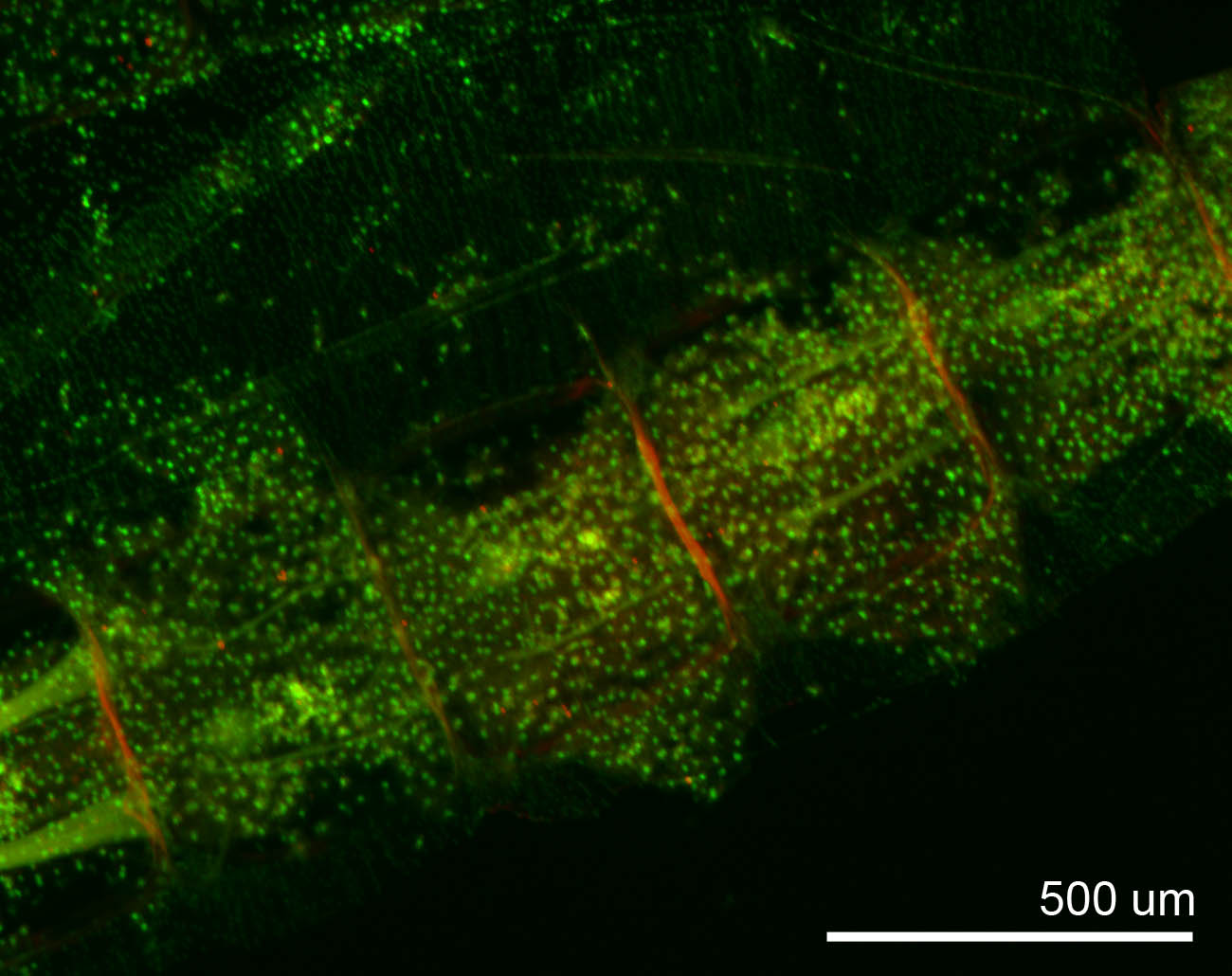


A

B

C

Supplementary Figure 3. FISH controls (as described in text). A) no probe control; B) competition control; C) RNase control
