# Supplementary material for: Wolbachia Infections Are Virulent and Inhibit the Human Malaria Parasite Plasmodium Falciparum in Anopheles Gambiae
Source: PLoS Pathog. 2011 May 19;7(5):e1002043. doi: 10.1371/journal.ppat.1002043 (PMC3098226; doi:10.1371/journal.ppat.1002043)
Supplement: Table S1 — PCR primers used in this study. (DOC) [file ppat.1002043.s005.doc]

Supplementary Table 1. PCR primers used in this study.

| Primer | Sequence 5'- 3' | Reference |
| --- | --- | --- |
| qHSC70B-F | GCGATCCAGGCCGACAT | (1) |
| qHSC70B-R | TCTTTGGCTTGCCCTCGAT | (1) |
| TEP15-F | TCGTCGCTGCTGGCAGTGTG |  |
| TEP15-R | AGGCCGGAGCAGTTTCGCAC |  |
| LRR-F | AAATTTGAACCGTCTCGCACATCT |  |
| LRR-R | TAGCCCGTTCACATCGAGTCTTA |  |
| FBN9-F | GAAATTGGCAGTGAGGCGGAGATG |  |
| FBN9-R | CCCCTTGTGGTACGTCAGCGAGTC |  |
| SRPN6-F | CGGTCAGTGGAATCCGGTACTACA | (2) |
| SRPN6-R | GCCGTACGCACCATTGGT | (2) |
| CLIP7A-F | CCTGGACAGCAAGGTGCGGG |  |
| CLIP7A-R | GGAGTTGGAACGCCTCCGGC |  |
| Cecropin-F | CCAGAGACCAACCAACCACC | (3) |
| Cecropin-R | GCACTGCCAGCACGACAAAGA | (3) |
| Cactus-F | GAACGGCTGCGCTTTAACA | (3) |
| Cactus-R | TCGTTCAAGTTCTGTGCAAGTGT | (3) |
| Rel1A-F | TCAACAGATGCCAAAAGAGGAAAT | (4) |
| Rel1A-R | CTGGTTGGAGGGATTGTG | (4) |
| Caspar-F | TCCACACATGCAACCTGTTT |  |
| Caspar-R | CTCGCTGCAGCACAGCGGTA |  |
| Rel2-F | ACCGATACGGAAAGTGTGCT | (5) |
| Rel2-R | CGGTGCTCCTCGTAATGACT | (5) |
| Pop WD_0550-F | CAGGAGTTGCTGTGGGTATATTAGC | (6) |
| Pop WD_0550-R | TGCAGGTAATGCAGTAGCGTAAA | (6) |
| Alb-GF | GGTTTTGCTTATCAAGCAAAAG | (7) * |
| Alb-GR | GCGCTGTAAAGAACGTTGATC | (7) * |
| S7-F | TCCTGGAGCTGGAGATGAAC | (8) |
| S7-R | GACGGGTCTGTACCTTCTGG | (8) |

* Primers modified from reference.
